# Association Between Maternal Folic Acid Supplementation and Congenital Heart Defects in Offspring in Birth Cohorts From Denmark and Norway

**DOI:** 10.1161/JAHA.118.011615

**Published:** 2019-03-12

**Authors:** Nina Øyen, Sjurdur F. Olsen, Saima Basit, Elisabeth Leirgul, Marin Strøm, Lisbeth Carstensen, Charlotta Granström, Grethe S. Tell, Per Magnus, Stein E. Vollset, Jan Wohlfahrt, Mads Melbye

**Affiliations:** ^1^ Department of Global Public Health and Primary Care University of Bergen Norway; ^2^ Department of Epidemiology Research Statens Serum Institut Copenhagen Denmark; ^3^ Department of Medical Genetics Haukeland University Hospital Bergen Norway; ^4^ Department of Heart Disease Haukeland University Hospital Bergen Norway; ^5^ Faculty of Natural and Health Sciences University of the Faroe Islands Tórshavn Faroe Islands; ^6^ Centre for Fertility and Health Norwegian Institute of Public Health Oslo Norway; ^7^ Department of Clinical Medicine University of Copenhagen Denmark; ^8^ Department of Medicine Stanford University School of Medicine Stanford CA

**Keywords:** congenital cardiac defect, folate, MoBa (Norwegian Mother and Child Cohort Study), pregnancy, prospective cohort study, Epidemiology, Pediatrics, Risk Factors, Cardiovascular Disease, Pregnancy

## Abstract

**Background:**

Evidence linking individual‐level maternal folic acid supplementation to offspring risk of congenital heart defects is lacking. We investigated whether folic acid supplementation in early pregnancy reduces offspring risk of heart defects in 2 large birth cohort studies.

**Methods and Results:**

Women recruited in early pregnancy within the DNBC (Danish National Birth Cohort), 1996–2003, and MoBa (Norwegian Mother and Child Cohort Study), 2000–2009, were followed until delivery. Information on periconceptional intake of folic acid and other supplements was linked with information on heart defects from national registers. Among 197 123 births, we identified 2247 individuals with heart defects (114/10 000). Periconceptional (4 weeks before through 8 weeks after conception) use of folic acid plus other supplements (54.8%), folic acid only (12.2%), and non–folic acid supplements (5.0%) were compared with no supplement use (28.0%); the adjusted relative risks of heart defects were 0.99 (95% CI, 0.80–1.22), 1.08 (95% CI, 0.93–1.25), and 1.07 (95% CI, 0.97–1.19), respectively. For initiation of folic acid in the preconception period weeks −4 to −1 (33.7%) and the postconception periods 0 to 4 weeks (15.5%), 5 to 8 weeks (17.8%), and 9 to 12 weeks (4.6%), compared with no or late folic acid intake (29.1%), relative risks of heart defect were 1.11 (95% CI, 1.00–1.25), 1.09 (95% CI, 0.95–1.25), 0.98 (95% CI, 0.86–1.12), and 0.97 (95% CI, 0.78–1.20), respectively. Relative risks of severe defects, conotruncal defects, and septal defects showed similar results.

**Conclusions:**

Folic acid was not associated with offspring risk of heart defects, including severe defects, conotruncal defects, or septal defects.

**Clinical Perspective**

**What Is New?**

- In 2 independent prospective birth cohorts of almost 200 000 births, we identified 2247 children with congenital heart defects. Our results do not support the hypothesis that maternal intake of supplements containing folic acid before or after conception reduces the risk of congenital heart defects in offspring.
- Our null association between maternal folic acid supplementation and offspring congenital heart defects was consistent across various definitions of folic acid exposure and types of cardiac defects.

**What Are the Clinical Implications?**

- Although most likely not harmful, the effect of maternal folic acid supplementation with respect to preventing cardiac birth defects may be questioned, at least in regions with sufficient intake of dietary folate.
- Our finding of no association between individual‐level maternal folic acid supplementation and offspring heart defect is in contrast to time trend studies claiming a causal relationship between folic acid and heart defects when comparing trends of birth prevalence of heart defects before and after folic acid fortification of staple food.

## Introduction

Congenital heart defects are the most common birth defects worldwide, but their etiology is largely unknown.^1^, ^2^ Gene defects contribute to the occurrence of heart malformations,^3^ but most heart defects occur in isolation in a family.^4^ Consequently, a large proportion of heart defects presumably arise in susceptible individuals who carry low‐penetrance genes or gene combinations, possibly in interaction with maternal or intrauterine factors. Although it is well established that maternal intake of folic acid around the time of conception reduces the risk of neural tube defect in offspring,^5^, ^6^ its association with reduced risk of congenital heart defects is only suggestive.^7^, ^8^

Although the precise role of folic acid supplementation on cardiac morphogenesis remains unclear, folic acid may have a role in the migration of the cardiac neural cells that contribute to the development of the embryonic heart.^9^, ^10^ It is important to corroborate this hypothesis with evidence from clinical and population‐based cohort studies with prospectively collected information on individual‐level maternal folic acid intake. To date, this hypothesis is supported by results from 1 randomized trial of high‐dose folic acid supplementation,^11^ 2 case–control studies^7^, ^8^ and 1 hospital‐based case–control study.^12^ However, no significant associations were found in 4 other case–control studies of birth defects including cardiac defects, in studies of cardiac outflow defects,^13^, ^14^, ^15^, ^16^ or in a nationwide cohort study of supplement use in pregnancy and offspring risk of heart defect.^17^ The inconsistencies among these studies may be related to design differences, recall bias of retrospectively collected exposure data in case–control studies, or less accurate information on dose or timing of folic acid supplementation in pregnancy; therefore, the evidence for a preventive effect of folic acid supplementation on heart defects is still inconclusive.

Knowledge of whether folic acid supplementation may reduce the risk of heart defects is important in strategies for the prevention of congenital malformations.^18^ Heart defects account for a third of infant deaths caused by malformations in industrialized countries,^19^ and severe heart defects require costly and complex surgical intervention.^20^ To date, Denmark and Norway have a policy of no food fortification with folic acid.

We have utilized 2 unique large prospective birth cohorts, the DNBC (Danish National Birth Cohort)^21^ and MoBa (Norwegian Mother and Child Cohort Study)^22^ to investigate whether periconceptional folic acid supplementation measured as individual‐level information of folic acid exposure from up to 4 weeks before through 8 weeks after conception, including the timing of folic acid initiation, reduces the risk of congenital heart defects.

## Methods

We can make tabulated data and analytical methods available to other researchers on reasonable request sent to the corresponding author.

### Independent Data Access and Analyses

All authors had the ability to query any aspect of the data either directly or through independent analysis. Authors N.Ø., S.B., and J.W. had full access to all data in the study and take responsibility for its integrity and the data analysis.

### Birth Cohorts

#### DNBC, 1996–2003

More than 100 000 Danish women in early pregnancy were recruited to the DNBC.^21^ By linkage, all births were updated with information on heart defects in their children, retrieved from the National Patient Register (NPR) and the Causes of Death Register, classified into cardiac phenotypes as published previously.^4^, ^23^ In addition, information on chromosomal aberrations from the Danish Cytogenetic Central Register, University of Aarhus, and familial heart defect cases from the Danish Familial Relational Database were included. The NPR contains information on in‐patient diagnoses assigned since 1977 and outpatient diagnoses from 1995 onward. Diagnoses of heart defects in the NPR have been validated against clinical records.^24^ The Causes of Death Register contains death certificate information, including underlying cause of death and up to 3 contributing causes of death since 1970, with high validity for infant death causes.^25^ The Danish Cytogenetic Central Register was established in 1968 and contains reports on all pre‐ and postnatal chromosomal analyses performed in Denmark since 1970 and 1960, respectively.

#### MoBa, 2000–2009

By June 2009, >100 000 pregnant women were included in MoBa.^22^ We used the MoBa analytic data file version 7, linked with information from the Medical Birth Registry of Norway. All births in MoBa were updated with information on heart defect from a national project database on heart defects, Congenital Heart Defects in Norway, Cardiovascular Disease in Norway Project (https://cvdnor.w.uib.no/), in which individuals with heart defects were ascertained from 4 data sources: the Medical Birth Registry of Norway, the hospitals’ patient administrative systems, Oslo University Hospital's clinical database for children with heart disease, and the Causes of Death Registry.^26^ Individuals with heart defects had been assigned specific cardiac phenotypes before linkage with MoBa. Information on chromosomal aberration and familial heart defect was ascertained from the Medical Birth Registry.

### Dietary Supplement: Assessment of Folic Acid Exposure

In the DNBC, information on folic acid brand names, dosage, and frequency of use was collected at enrollment covering the period from 4 weeks before the last menstrual period until enrollment (around the 12th gestational week). At enrollment the women answered a questionnaire including information on supplement use. This recruitment form had 2 formats during the study period: initially a format with a floating time window and then, halfway through the study period, a format with a fixed time window (each week specified from gestational week −4 to week 14). Information from the first recruitment form was manually set to the same format as the second recruitment form.^27^ In MoBa, the women answered a questionnaire including information on folic acid supplement use and intake of specific nutrients from 3 months before pregnancy and throughout pregnancy until enrollment (18th gestational week). More detailed information on exposure matrixes in both cohorts has been published.^28^, ^29^, ^30^ Data have also been used to evaluate health campaigns for the intake of folic acid during pregnancy.^29^ Self‐reported folic acid intake has been validated and found to mirror biological levels of folate in erythrocytes^31^ and in plasma.^32^ Therefore, use of folic acid is one of the most well‐defined exposures so far characterized collaboratively between the 2 cohorts with the original objective of studying its influence on neural tube defects.

The embryonic heart forms from the second through the eighth weeks after conception.^33^ Therefore, the presumed window of susceptibility from insufficient maternal folate regarding embryonic heart development was defined as 4 weeks before through 8 weeks after conception. Exposure information of maternal folic acid supplement use was classified by 2 approaches (supplement type and timing), as suggested by Roth and colleagues.^34^, ^35^ First, we assessed any supplement use 4 weeks before through 8 weeks after conception for 4 mutually exclusive exposure categories: folic acid (0.4 mg) plus other micronutrient supplements, only folic acid, other non–folic acid supplements, and no supplements (as the reference). Next, we evaluated initiation of any folic acid intake (folic acid plus other supplements were combined with folic acid only) for 4 time windows: 4 weeks before conception, 4 weeks after conception, 5 to 8 weeks after conception, and 9 to 12 weeks (9–11 weeks in Denmark) after conception, with no folic acid supplementation (no supplements was combined with non–folic acid supplements) in the period 4 weeks before to 12 weeks (11 weeks in Denmark) after conception (as the reference).

### Case Ascertainment and Classification of Congenital Heart Defects

In both birth cohorts, infants with *International Classification of Diseases, 10th Revision (ICD‐10)* codes for heart defects were classified into cardiac phenotypes in a hierarchical system^23^, ^26^, ^36^: heterotaxia, conotruncal defects, atrioventricular septal defects, anomalous pulmonary venous return, left ventricle outflow tract obstruction, right ventricle outflow tract obstruction, isolated septal defects (ventricular septal defects, atrial septal defects registered after 6 weeks, and ventricular plus atrial septal defects), complex defects, patent ductus arteriosus registered after 6 weeks or with surgical correction in preterm live births (gestational age <37 weeks) or live births at term (gestational age ≥37 weeks), other specified heart defects, unspecified heart defects (Table S1). In tables, we report relative risk estimates for any type of heart defect (not counting patent ductus arteriosus in preterm births), severe heart defects (heterotaxia, conotruncal defects, atrioventricular septal defects, anomalous pulmonary venous return, left or right ventricle outflow tract obstruction, complex defects combined), conotruncal defects, and septal defects (ventricular septal defects, atrial septal defects registered after 6 weeks, and ventricular plus atrial septal defects).

### Potential Confounders and Stratification Variables

We decided a priori on the following potential confounders (variables associated with maternal folic acid supplement use and most likely also with the risk of heart defect in offspring) for the association between maternal folic acid supplementation and offspring heart defect risk: year of birth and birth order, maternal age, prepregnancy body mass index (kg/m^2^), and epilepsy (or intake of folic acid antagonists for maternal epilepsy). In addition, we also considered maternal socioeconomic status and education, pregestational diabetes mellitus, smoking, and alcohol consumption as possible confounders. We did not, however, include these factors in the final model because they were either associated only with the exposure (maternal education, smoking, alcohol consumption) or only with heart defects in the offspring (pregestational diabetes mellitus). We also explored the main effects when restricting the analyses by information on live births, singletons, mother without heart defect, or planned pregnancy. Individuals with missing data for the covariates among all births (10 352 of 197 213, 5.5%) and births with cardiac defect (117 of 2247, 5.2%) were not included in the multivariable regression analyses.

### Study Cohort and Statistical Analysis

The 2 study cohorts consisted of live births, and MoBa also included stillbirths and pregnancy termination for fetal abnormalities. We excluded births with chromosomal aberrations (353 in DNBC and 140 births in MoBa). The association between maternal folic acid intake and the risk of heart defect was calculated as risk ratios (RRs) comparing offspring risk of heart defect among those exposed and not exposed to supplementation. The RRs were estimated with 95% CIs in log‐linear binominal regression analyses with adjustment for the a priori confounders in the separate analyses of the Danish and Norwegian cohorts, and then with adjustment for interactions between country and the a priori country‐specific confounders when combining the 2 cohorts.

### Study Size and Power Calculation

A priori, we estimated that we would be able to detect a reduced risk of heart defect (all types combined) and conotruncal defects among infants exposed to folic acid supplementation of at least 16% and 41%, respectively, relative to unexposed infants. Estimates were based on 80% power and a 5% significance level, the Danish prevalence of heart defects without chromosomal aberrations in 93 per 10 000 and in 12 per 10 000 for conotruncal defects,^23^ and a mean prevalence of folic acid supplementation of 23% (Denmark 13.5%^37^ and Norway 32.0%^28^).

### Ethics

We have approval from the DNBC's steering committee (ref. no 2010‐05; May 4, 2010), a general approval granted to the Statens Serum Institut from the Danish Data Protection Agency (J.nr.2008‐54‐0472; September 12, 2008), and an approval from the steering board of the Danish Cytogenetic Central Register. We also have approval from MoBa's steering committee (PD757; 09/3237; February 5, 2010); the Regional Committee for Medical and Health Research Ethics, South‐East Norway (2012/796); and the steering committee of the Cardiovascular Disease in Norway project. Written informed consent was obtained from all participating women.

## Results

### Denmark: DNBC

In the study cohort from Denmark of 94 228 births without chromosomal aberrations, we present information on mothers’ periconceptional intake of supplements (Table 1). In the period 1996–2003, maternal intake of folic acid plus other supplements not containing folic acid was reported in 59.0% of the births, intake of only folic acid was reported in 4.1%, use of other non–folic acid supplements was reported in 3.7%, and no maternal supplement intake was reported in 33.2%.

**Table 1:** Maternal Characteristics by Maternal Use of Supplements From 4 Weeks Before to 8 Weeks After Conception Among 94 228 Births Without Chromosomal Aberrations in the DNBC, Denmark, 1996–2003

Characteristics	TotalN=94 228 (100%)	No Supplementsn=31 322 (33.2%)	Other Supplements No Folic Acidn=3453 (3.7%)	Folic Acid Onlyn=3889 (4.1%)	Folic Acid Plus Other Supplementsn=55 564 (59.0%)	*P* Value, No Folic Acid vs Folic Acid Use^a^
Year of birth^b^						
1996	139 (0.15)	69 (49.6)	12 (8.6)	0 (0)	58 (41.7)	<0.0001
1997	750 (0.80)	346 (46.1)	43 (5.7)	9 (1.2)	352 (46.9)	
1998	12 077 (12.8)	4442 (36.8)	767 (6.4)	260 (2.2)	6608 (54.7)	
1999	18 548 (19.7)	7188 (38.8)	924 (5.0)	459 (2.5)	9977 (53.8)	
2000	20 540 (21.8)	7230 (35.2)	764 (3.7)	823 (4.0)	11 723 (57.1)	
2001	20 135 (21.4)	6254 (31.1)	555 (2.8)	933 (4.6)	12 393 (61.6)	
2002	18 084 (19.2)	4681 (25.9)	324 (1.8)	1123 (6.2)	11 956 (66.1)	
2003	3955 (4.2)	1112 (28.1)	64 (1.6)	282 (7.1)	2497 (63.1)	
Maternal age, y^b^						
≤24	8996 (9.55)	3871 (43.0)	343 (3.8)	287 (3.2)	4495 (50.0)	<0.0001
25–29	35 891 (38.1)	11 515 (32.1)	1187 (3.3)	1488 (4.2)	21 701 (60.5)	
30–34	35 117 (37.3)	11 277 (32.1)	1279 (3.6)	1479 (4.2)	21 082 (60.0)	
≥35	14 113 (15.0)	4628 (32.8)	640 (4.5)	627 (4.4)	8218 (58.2)	
Missing	111 (0.12)	31 (27.9)	4 (3.6)	8 (7.2)	68 (61.3)	
Birth order						
First	41 698 (44.3)	12 255 (29.4)	1374 (3.3)	2049 (4.9)	26 020 (62.4)	<0.0001
Second	32 425 (34.4)	10 829 (33.4)	1226 (3.8)	1188 (3.7)	19 182 (59.2)	
Third	11 435 (12.1)	4542 (39.7)	497 (4.4)	384 (3.4)	6012 (52.6)	
Fourth or higher	2649 (2.8)	1171 (44.2)	146 (5.5)	76 (2.9)	1256 (47.4)	
Missing	6021 (6.4)	2525 (41.9)	210 (3.5)	192 (3.2)	3094 (51.4)	
Maternal body mass index before pregnancy (kg/m^2^)^b^						
Single	90 180 (95.7)	30 261 (33.6)	3318 (3.7)	3716 (4.1)	52 885 (58.6)	<0.0001
Twins/triplets	4048 (4.3)	1061 (26.2)	135 (3.3)	173 (4.3)	2679 (66.2)	

Data are shown as n (%). DNBC indicates Danish National Birth Cohort.

a No folic acid use (no/other supplements, no folic acid) vs folic acid use (folic acid only/folic acid plus other supplements) 4 weeks before to 8 weeks after gestation (combining −4 to 0, 0–4, and 5–8 weeks).

b Data from Medical Birth Registry.

### Norway: MoBa

The study cohort from Norway consisted of 102 985 births without chromosomal aberrations (Table 2). In the period 1999–2009, the mother reported intake of folic acid plus other supplements in 50.3% of the births, folic acid only in 19.0%, other supplements without folic acid in 6.2%, and no supplement intake in 24.5%.

**Table 2:** Maternal Characteristics by Maternal Use of Supplements From 4 Weeks Before to 8 Weeks After Conception Among 102 985 Births Without Chromosomal Aberrations in MoBa, Norway, 1999–2009

Characteristics	TotalN=102 985 (100%)	No Supplementsn=25 229 (24.5%)	Other Supplements No Folic Acidn=6431 (6.24%)	Folic Acid Onlyn=19 555 (19.0%)	Folic Acid Plus Other Supplementsn=51 770 (50.3%)	*P* Value, No Folic Acid vs Folic Acid use^a^
Year of birth^b^						
1999	46 (0.04)	30 (65.2)	4 (8.7)	6 (13.0)	6 (13.0)	<0.0001
2000	2010 (2.0)	1047 (52.1)	223 (11.1)	316 (15.7)	424 (21.1)	
2001	3931 (3.8)	1890 (48.8)	443 (11.3)	609 (15.5)	989 (25.2)	
2002	8293 (8.1)	3960 (47.8)	846 (10.2)	1321 (15.9)	2166 (26.1)	
2003	12 142 (11.8)	4615 (38.0)	1133 (9.3)	2208 (18.2)	4186 (34.5)	
2004	13 073 (12.7)	3282 (25.1)	845 (6.5)	2541 (19.4)	6405 (49.0)	
2005	15 117 (14.7)	3181 (21.0)	785 (5.2)	3094 (20.5)	8057 (53.3)	
2006	16 775 (16.3)	2872 (17.1)	867 (5.2)	3177 (18.9)	9859 (58.8)	
2007	15 474 (15.0)	2265 (14.6)	661 (4.3)	3028 (19.6)	9520 (61.5)	
2008	12 904 (12.5)	1695 (13.1)	514 (4.0)	2620 (20.3)	8075 (62.6)	
2009	3220 (3.1)	392 (12.2)	110 (3.4)	635 (19.7)	2083 (64.7)	
Maternal age, y^b^						
≤24	11 301 (11.0)	3999 (35.4)	883 (7.8)	2011 (17.8)	4408 (39.0)	<0.0001
25–29	33 730 (32.8)	7920 (23.5)	1846 (5.5)	6739 (20.0)	17 225 (51.1)	
30–34	39 916 (38.8)	8881 (22.3)	2378 (6.0)	7772 (19.5)	20 885 (52.3)	
≥35	18 038 (17.5)	4429 (24.6)	1324 (7.3)	3033 (16.8)	9252 (51.3)	
Birth order^b^						
First	46 367 (45.0)	9566 (20.6)	2723 (5.9)	8282 (17.9)	25 796 (55.6)	<0.0001
Second	36 576 (35.5)	8936 (24.4)	2105 (5.8)	7616 (20.8)	17 919 (49.0)	
Third or higher	20 042 (19.5)	6727 (33.6)	1603 (8.0)	3657 (18.3)	8055 (40.2)	
Maternal body mass index before pregnancy (kg/m^2^)^b^						
<20	12 700 (12.3)	2953 (23.3)	875 (6.9)	2104 (16.6)	6768 (53.3)	<0.0001
20–24	56 079 (54.5)	12 656 (22.6)	3592 (6.4)	10 450 (18.6)	29 381 (52.4)	
25–29	21 735 (21.1)	5671 (26.1)	1273 (5.9)	4505 (20.7)	10 286 (47.3)	
30–24	7067 (6.9)	2154 (30.5)	366 (5.2)	1442 (20.4)	3105 (43.9)	
≥35	2608 (2.5)	784 (30.1)	132 (5.1)	553 (21.2)	1139 (43.7)	
Missing data	2796 (2.7)	1011 (36.2)	193 (6.9)	501 (17.9)	1091 (39.0)	
Maternal heart defect						
Yes	640 (0.62)	160 (25.0)	48 (7.5)	103 (16.1)	329 (51.4)	0.20
No	102 345 (99.4)	25 069 (24.5)	6383 (6.2)	19 452 (19.0)	51 441 (50.3)	
Epilepsy before pregnancy						
Yes	687 (0.67)	160 (23.3)	28 (4.1)	140 (20.4)	359 (52.3)	0.08
No	102 298 (99.3)	25 069 (24.5)	6403 (6.3)	19 415 (19.0)	51 411 (50.3)	
Prepregnancy diabetes mellitus^c^						
Yes	698 (0.68)	172 (24.6)	40 (5.7)	123 (17.6)	363 (52.0)	0.70
No	102 287 (99.3)	25 057 (24.5)	6391 (6.25)	19 432 (19.0)	51 407 (50.2)	
Smoking before pregnancy						
Daily	18 598 (18.1)	6574 (35.4)	1318 (7.1)	3438 (18.5)	7268 (39.1)	<0.0001
Sometimes	10 309 (10.0)	2388 (23.2)	628 (6.1)	1917 (18.6)	5376 (52.2)	
No	61 352 (59.6)	11 346 (18.5)	3315 (5.4)	11 929 (19.4)	34 762 (56.7)	
Missing	12 726 (12.4)	4921 (38.7)	1170 (9.2)	2271 (17.9)	4364 (34.3)	
Prepregnancy alcohol intake^d^						
Yes	88 509 (85.9)	16 824 (20.4)	4635 (5.61)	16 027 (19.4)	45 149 (54.6)	<0.0001
No	7167 (6.96)	1459 (34.0)	363 (8.46)	785 (18.3)	1686 (39.3)	
Missing	7309 (7.10)	1878 (44.7)	419 (9.97)	702 (16.7)	1205 (28.7)	
Maternal education, y						
<12	20 535 (19.9)	7987 (38.9)	1648 (8.03)	3661 (17.8)	7239 (35.3)	<0.0001
12	14 458 (14.0)	4393 (30.4)	1081 (7.48)	2717 (18.8)	6267 (43.4)	
13–16	39 916 (38.8)	8021 (20.1)	2229 (5.58)	8071 (20.2)	21 595 (54.1)	
≥17	22 816 (22.2)	3337 (14.6)	1116 (4.89)	4166 (18.3)	14 197 (62.2)	
Missing data	5260 (5.11)	1491 (28.4)	357 (6.79)	940 (17.9)	2472 (47.0)	
Planned pregnancy						
Yes	82 078 (79.7)	18 144 (22.1)	4664 (5.68)	16 377 (20.0)	42 893 (52.3)	<0.0001
No	19 673 (19.1)	6658 (33.8)	1672 (8.50)	2997 (15.2)	8346 (42.4)	
Missing data	1234 (1.20)	427 (34.6)	95 (7.70)	181 (14.7)	531 (43.0)	
Plurality^b^						
Single	99 320 (96.4)	24 450 (24.6)	6226 (6.3)	18 800 (18.9)	49 844 (50.2)	<0.0001
Twins/triplets	3665 (3.6)	779 (21.3)	205 (5.6)	755 (20.6)	1926 (52.6)	

Data are shown as n (%). MBRN indicates Medical Birth Registry of Norway; MoBa, Norwegian Mother and Child Cohort Study.

a No folic acid use (no/other supplements, no folic acid) vs folic acid use (folic acid only/folic acid plus other supplements) 4 weeks before to 8 weeks after gestation (combining −4 to 0, 0–4, and 5–8 weeks).

b Numbers from MBRN.

c Diabetes mellitus type 1 or type 2 before pregnancy, registered in MoBa and/or MBRN.

d Alcohol intake 3 months before pregnancy.

Maternal use of folic acid or other supplements is shown for maternal and birth characteristics in both cohorts (Tables 1 and 2).

### Cardiac Defects and Birth Prevalence in the Danish and Norwegian Birth Cohorts

Country‐specific numbers of heart defects and birth prevalence are presented in Table 3. The 2 birth cohorts were combined, and among 197 213 births, there were 2247 infants with any type of heart defect, not counting patent ductus arteriosus among preterm births (birth prevalence 114 per 10 000 births). There were 583 infants with severe heart defect (30 per 10 000), of which 201 had conotruncal defect (10 per 10 000 births), and 1191 infants had septal defect (60 per 10 000).

**Table 3:** Numbers and Birth Prevalence of Congenital Heart Defects Among 94 228 Births in the DNBC, 1996–2003, and Among 102 985 Births in MoBa, Norway, 2000–2009

	Denmark (n=94 228)		Norway (n=102 985)	
	n	n/10 000	n	n/10 000
Any heart defect	1077	114	1434	139
Any heart defect without preterm PDA	995	106	1252	122
Heterotaxia	16	1.7	13	1.3
Conotruncal defect	105	11	96	9.3
AVSD	34	3.6	27	2.6
APVR	8	0.8	12	1.2
LVOTO	96	10	84	8.2
RVOTO	43	4.6	48	4.7
Septal defect, isolated	445	47	746	72
VSD	235	25	586	60
ASD	141	15	138	13
VSD+ASD	34	3.6	12	1.2
Unspecified septal defect	35	3.7	10	1.0
Other complex defects	1	0.1	0	
Isolated PDA^a^	155	16	279	27
At‐term gestation	73	7.7	97	9.4
Preterm gestation	82	8.7	182	18
Other specified defects	87	9.2	91	8.8
Unspecified cardiac defects	87	9.2	38	3.7

APVR indicates anomalous pulmonary venous return; ASD, atrial septal defect; AVSD, atrioventricular septal defect; DNBC, Danish National Birth Cohort; LVOTO, left ventricle outflow tract obstruction; MoBa, Norwegian Mother and Child Cohort Study; PDA, patent ductus arteriosus; RVOTO, right ventricle outflow tract obstruction; VSD, ventricular septal defect.

a Isolated PDA in preterm infants (gestational age <37 weeks) was not included in the regression analyses.

### Folic Acid Supplementation and Risk of Congenital Heart Defects in Denmark and Norway

Excluding 5.2% with missing values, the combined cohort consisted of 186 861 births. In the period from 4 weeks before through 8 weeks after conception, 54.8% of the mothers used folic acid plus other types of non–folic acid supplements, 12.2% took only folic acid supplements, 5.0% used non–folic acid supplements, and 28.0% did not use any supplements (Table 4). The adjusted RRs for any type of heart defect in offspring were 0.99 (95% CI, 0.80–1.22) for maternal intake of folic acid plus other supplements, 1.08 (95% CI, 0.93–1.25) for only folic acid supplements, and 1.07 (95% CI, 0.97–1.19) for non–folic acid supplements, compared with no supplement use. RR estimates were similarly nonsignificant for conotruncal defects, severe heart defects, and septal defects for any combination of intake of folic acid and non–folic acid supplementation.

**Table 4:** Relative Risk of Congenital Heart Defect^a^ (Overall), Severe Heart Defect^b^, Conotruncal Defect,^c^ and Septal Defect^d^ by Maternal Intake of Folic Acid Supplements in the Periconceptional Period, Combining 94 228 Births in the DNBC, Denmark, 1996–2003, and 102 985 Births in MoBa, Norway 1999–2009

	Total Births, N=186 861^e^	Any Congenital Heart Defect,^a^n=2130			Severe Heart Defect,^b^n=551			Conotruncal Heart Defect,^c^n=187			Septal Defect,^d^n=1139		
	n (%)	n	Adjusted RR^f^(95% CI)		n	Adjusted RR^f^(95% CI)		n	Adjusted RR^f^(95% CI)		n	Adjusted RR^f^(95% CI)	
Supplement use (4 wk before to 8 wk after conception)													
None	52 401 (28.0)	569	1	Reference	157	1	Reference	55	1	Reference	296	1	Reference
Other supplements, no folic acid	9414 (5.0)	104	0.99	0.80–1.22	25	0.92	0.61–1.41	8	0.84	0.40–1.77	61	1.06	0.80–1.39
Folic acid only	22 695 (12.2)	279	1.08	0.93–1.25	68	1.10	0.82–1.48	22	0.98	0.59–1.64	155	1.03	0.84–1.26
Folic acid plus other supplementation	102 351 (54.8)	1178	1.07	0.97–1.19	301	1.02	0.84–1.25	102	0.93	0.66–1.31	627	1.06	0.92–1.22
Initiation of folic acid^g^ (4 wk before to 12 wk after conception)													
None	53 142 (28.4)	577	1	Reference	158	1	Reference	57	1	Reference	301	1	Reference
Weeks −4 to −1	62 924 (33.7)	753	1.11	1.00–1.25	192	1.08	0.87–1.34	65	0.95	0.66–1.38	397	1.08	0.92–1.26
Weeks 0–4	28 961 (15.5)	348	1.09	0.95–1.25	82	1.01	0.77–1.33	29	0.93	0.59–1.48	199	1.11	0.92–1.33
Weeks 5–8	33 161 (17.8)	356	0.98	0.86–1.12	95	1.00	0.77–1.30	30	0.85	0.54–1.33	186	0.93	0.77–1.11
Weeks 9–12^h^	8673 (4.6)	96	0.97	0.78–1.20	24	0.97	0.63–1.50	6	0.68	0.29–1.58	56	1.00	0.75–1.32

APVR indicates anomalous pulmonary venous return; ASD, atrial septal defect; AVSD, atrioventricular septal defect; DNBC, Danish National Birth Cohort; LVOTO, left ventricle outflow tract obstruction; MoBa, Norwegian Mother and Child Cohort Study; PDA, patent ductus arteriosus; RR, risk ratio; RVOTO, right ventricle outflow tract obstruction; VSD, ventricular septal defect.

a Congenital heart defects: Heterotaxia, conotruncal defects, AVSD, APVR, LVOTO, RVOTO, VSD, ASD, VSD plus ASD, complex defects; patent ductus arteriosus (PDA) in live births at term (gestational age ≥37 weeks); other specified heart defects; unspecified heart defects.

b Severe heart defects: heterotaxia, conotruncal defects (see below), AVSD, APVR, LVOTO, RVOTO, complex defects.

c Conotruncal defects: truncus arteriosus, transposition of the great arteries, tetralogy of Fallot, pulmonary atresia with ventricular septal defect (tetralogy of Fallot type), double‐outlet right ventricle, conoventricular septal defect, interrupted aortic arch type B or C, supravalvular aortic stenosis, aortopulmonary window.

d Septal defects: VSD, ASD, VSD plus ASD.

e Individuals with missing values of covariates were excluded in the adjusted analyses (total births n=10 352 [5.2% of 197 213]; any heart defect n=117 [5.2% of 2247]; severe defects n=32; conotruncal defects n=14; septal defects n=52); see country‐specific numbers in Tables S2 and S3.

f Relative risk with 95% CI comparing supplement use with no use (reference) 4 weeks before to 8 weeks after conception (upper panel) or comparing initiation of folic acid supplements with no use or non–folic acid supplements (reference) 4 weeks before to 12 weeks after conception (lower panel). RRs adjusted for interaction between country (Denmark/Norway) and the following factors: year of birth (Denmark: 1996–1997, 1998, 1999, 2000, 2001, 2002–2003; Norway: 1999–2000, 2001, 2002, 2003, 2004, 2005, 2006, 2007, 2008–2009), maternal age (≤24, 25–29, 30–34, ≥35 years), birth order (first, second, third or higher), maternal body mass index (<20, 20–24, 25–29, 30–34, ≥35), maternal heart defect (yes/no), maternal epilepsy before pregnancy (yes/no). Categories for adjustment variables combined for severe and conotruncal defects.

g The 4 exposure categories in supplement use collapsed into no/yes; *no* is no folic acid use (no supplements and other supplements, no folic acid), and *yes* is folic acid use (folic acid only and folic acid plus other supplementation.

h In DNBC, including week 11.

We next analyzed initiation of any folic acid supplementation within the period from 4 weeks before to 12 weeks after conception (Table 4). Among the 186 861 births, 33.7% of the mothers started folic acid supplementation during weeks −4 to −1, 15.3% started during weeks 0 to 4, 17.6% started during weeks 5 to 8, and 4.6% started during weeks 9 to 12, whereas 29.1% did not take folic acid supplementation or started to take folic acid supplementation after week 12. The adjusted RRs of any heart defect in offspring were 1.11 (95% CI, 1.00–1.25), 1.09 (95% CI, 0.95–1.25), 0.98 (95% CI, 0.86–1.12), and 0.97 (95% CI, 0.78–1.20) for maternal folic acid intake in weeks −4 to −1, 0 to 4, 5 to 8, and 9 to 12, respectively, compared with the reference group with no or late folic acid intake. RR estimates were similarly nonsignificant for severe heart defects, conotruncal defects, and septal defects for any timing of folic acid use compared with no folic acid use.

Numbers and relative risks of offspring heart defect for maternal intake of folic acid and other non–folic acid supplements were estimated separately for Denmark and Norway (Tables S2 and S3). In each country, the relative risk of heart defects in offspring was nonsignificant when comparing folic acid with no folic acid in early pregnancy.

In Table S4, adjusted RRs in the combined cohort showed similar null findings for the association between folic acid and any heart defect or conotruncal defects using the final model, model 2 with further adjustment for maternal education, or model 3 with the following covariates added to model 1: maternal diabetes mellitus, maternal smoking before conception, and maternal alcohol consumption 3 months before conception.

In sensitivity analyses, we restricted the combined cohort to live births, singleton births, births to mothers who did not have a heart defect, or births of a planned pregnancy, and we estimated sets of RRs for offspring risk of any heart defect (Table S5). The RRs showed no association between folic acid and any heart defect regardless of various restrictions of the birth cohort.

## Discussion

In 2 independent national birth cohorts in Denmark and Norway of almost 200 000 pregnancies with prospectively collected information on folic acid supplement intake with follow‐up of all births, we identified 2247 births with congenital heart defects in the offspring. We did not find support for the hypothesis that maternal periconceptional supplementation containing folic acid would reduce the risk of congenital heart defects. The findings were consistent using 2 exposure approaches, any intake of folic acid supplementation during the period from 4 weeks before and 8 weeks after conception, and timing of such intake in the periconceptional period. Specifically the risks of conotruncal defects, severe heart defects, or septal defects were not reduced by such supplementation.

### Previous Findings From Studies of the Association Between Folic Acid and Congenital Heart Defects

There are studies comparing birth prevalence trends before and after staple food folic acid fortification,^38^, ^39^ claiming a causal relationship between folic acid and heart defect.^38^ However, a prerequisite for discussion of causal inference are studies designed with individual‐level information of maternal folic acid supplement use and infant risk of heart defect. Although several studies have individual‐level information of folic acid intake, these studies have conflicting findings most likely related to different design, bias, or confounding, they could also be explained by regional differences in dietary vitamin insufficiency. A detailed discussion of previous reports in the context of regions—for example, the United States,^7^, ^15^, ^16^, ^40^, ^41^, ^42^ Hungary,^11^, ^43^ Western Australia,^14^ the Netherlands,^8^, ^13^ China,^12^ and Norway^17^—can be found in Data S1.

To summarize previous findings from studies of the association between folic acid and congenital heart defects, we found 1 randomized controlled trial reporting a nonsignificant protective effect of high‐dose folic acid supplements,^11^ whereas 1 cohort study^17^ and most other case–control studies^7^, ^13^, ^14^, ^15^, ^16^, ^40^, ^42^ except one of the Dutch studies^8^ do not support the hypothesis that folic acid supplementation prevents congenital heart defects. The overall lack of a protective effect from folic acid supplementation for heart defect risk in previous studies supports the null findings in the present study and is in stark contrast to inference drawn from trend studies of birth defects, including heart defects, before‐and‐after folic acid fortification, or folic acid recommendation to fertile women.^38^, ^44^

In support of the null findings in the present birth cohort study from Norway and Denmark, in particular for conotruncal defects, is a report using a Mendelian randomization approach that genotyped >3000 individuals with conotruncal defects for the variants in the *MTHFR* (methylenetetrahydrofolate reductase) gene.^45^ The variant 677TT has previously been found to be associated with reduced folate levels in blood and could be associated with offspring risk of heart defects. The multicenter study found no association between the variant 677TT and the risk of conotruncal defects compared with healthy controls. The authors also performed a meta‐analysis of 16 published studies on the relationship between conotruncal heart defects and the variant 677TT and found a weak positive association, likely explained by publication bias: namely, that the studies with nonsignificant or null associations are less frequently published.

### Interpretation of the Null Association Between Periconceptional Folic Acid and Cardiac Defects

There could be several explanations for the null findings between maternal folic acid intake in the periconceptional period and offspring risk of cardiac malformations in this study. First, the officially recommended folic acid dose of 0.4 mg/d could be insufficient to prevent cardiac malformations. The feature of the 2 studies from Hungary,^11^, ^43^ although of less optimal design, was the use of high‐dose folic acid (0.8 and 5.6 mg/d), which was associated with reduced risk of cardiac malformation. Second, the null findings in the present study could also be explained by sufficient prepregnancy folate status among the participating mothers. In less advantaged regions or countries with lower baseline folate levels among fertile women, folic acid supplementation could be beneficial for the prevention of cardiac defects, as has been suggested in parts of China.^12^ Third, mothers who take folic acid supplements are in general healthier than nonusers, indicated by lower body mass index, less smoking, more pregnancy planning, higher age at delivery, lower parity, or higher education,^32^ with some of these factors also reported to be associated with lower offspring risk of cardiac malformation. However, we were able to adjust for potentially negative confounding. Fourth, the timing when women start taking folic acid may be too late in the periconceptional period to prevent development of cardiac malformations. However, initiation of folic acid classified in 4‐week intervals in the period 4 weeks before through 11 to 12 weeks after conception all showed a null association with offspring risk of cardiac malformation. Fifth, it could be that folic acid supplements prevent neural tube defects^5^, ^6^ but not cardiac malformations. Maternal folate insufficiency may act differently during different time windows in embryonic organ development; folate insufficiency probably disturbs neural tube closure corresponding to an early period in embryo formation but not formation of the later embryonic heart development corresponding to a somewhat later period. Sixth, there could be nondifferential misclassification or measurement error of folic acid exposure and timing, which may bias the estimate to the null value. Interestingly, we found similar nonsignificant country‐specific relative risks despite differences in collection and classification of folic acid intake.

Although most likely not harmful,^46^, ^47^ the effect of maternal folic acid supplementation with respect to preventing cardiac birth defects may be questioned, at least in regions with sufficient intake of dietary folate.

### Study Strengths and Limitations

The use of prospectively collected information on individual‐level folic acid intake in early pregnancy precluded recall bias of exposure. We have complete follow‐up of all births (except for 204 mothers who gave birth abroad) through the nationwide Medical Birth Registers. Before the analyses, we used an established method for classifying folic acid exposure by type (folic acid plus other supplements, only folic acid, non–folic supplements, no supplements) in the period from 4 weeks before through 8 weeks after conception and by timing (initiation of supplement use) in the period from 4 weeks before through 11 to 12 weeks after conception,^34^, ^35^ corresponding to fetal cardiac development (third to eighth weeks after conception). Folic acid is a well‐characterized exposure in the 2 cohorts^27^, ^28^, ^48^ and is found to mirror blood plasma folate.^49^ We did not have information on perigestational dietary folate, but from previous studies, supplemental folic acid outweighs dietary folate, as women reporting folic acid supplement use have higher plasma folate levels than nonusers regardless of dietary intake.^32^

Low ascertainment of severe heart defects, including conotruncal defects, is unlikely because these defects almost always come to medical attention, either because of the need for surgery or at death. In Denmark there is good agreement between severe heart defect diagnoses in the NPR compared with hospital records,^24^ and in Norway, nationwide health registries and databases, as well as a clinical register, were combined to ensure virtually complete ascertainment of all heart defects among live births, stillbirths, and terminated pregnancies, including information on chromosomal aberrations and familial heart defect cases. All cases from the clinical register at Oslo University Hospital have been coded by senior pediatric cardiologists.

By combining country‐specific findings in 2 national birth cohorts, our study cohort was of sufficient size to produce narrow CIs, although we could not estimate RRs for the very rare outcomes of severe defects. A large set of potential confounders were available for evaluation, for adjustment or restriction of the study population for sensitivity analyses. Finally, although exposure data were collected somewhat differently in the 2 cohorts, we decided not to harmonize exposure among the 2 birth cohorts.^50^ The combined country‐specific estimates with corresponding country‐specific confounders illustrate that despite the variation in exposure collection and country‐specific birth prevalence of heart defect, we found no significant association between maternal folic acid supplementation and heart defect risk in either birth cohort.

In conclusion, maternal periconceptional use of folic acid supplements was not associated with offspring risk of congenital heart defects, including conotruncal defects or other severe defects, as well as septal defects, after combining 2 national birth cohorts with prospectively collected information of periconceptional supplement intake. The possible effect of maternal folic acid supplementation with respect to preventing cardiac birth defects may be questioned, at least in regions with sufficient intake of dietary folate.

## Sources of Funding

Dr Øyen was funded by grants from Lundbeck Foundation (R48‐A4793), Copenhagen, Denmark; Western Norway Regional Health Authorities (911734); Research Council Norway (190858/V50), and University of Bergen (Småforsk), Norway. Dr Olsen was funded by Innovation Fund Denmark (grant no 09‐06124, “Centre for Fetal Programming”). The DNBC (Danish National Birth Cohort) was established with a significant grant from the Danish National Research Foundation. Additional support was obtained from the Danish Regional Committees, the Pharmacy Foundation, the Egmont Foundation, the March of Dimes Birth Defects Foundation, the Health Foundation, and other minor grants. MoBa (Norwegian Mother and Child Cohort Study) is supported by the Norwegian Ministry of Health and Care Services and the Ministry of Education and Research and the US National Institutes of Health, National Institute of Neurological Disorders and Stroke (grant no. 1 UO1 NS 047537‐01 and grant no. 2 UO1 NS 047537‐06A1).

## Disclosures

None.

## Supporting information


**Data S1.** A detailed discussion on previous findings from studies of the association between folic acid and congenital heart defects.
**Table S1.** The Classification System for Congenital Heart Defect Phenotypes
**Table S2.** Relative Risk of Congenital Heart Defect* (Overall), Severe Heart Defect^†^, Conotruncal Defect^‡^, and Septal Defect^§^ by Maternal Intake of Folic Acid Supplements in the Periconceptional Period Among 94 228 Births the DNBC (Danish National Birth Cohort), Denmark, 1996–2003
**Table S3.** Relative Risk of congenital Heart Defect* (Overall), Severe Heart Defect^†^, Conotruncal Defect^‡^, and Septal Defect^§^ by Maternal Intake of Folic Acid Supplements in the Periconceptional Period Among 102 985 Births in MoBa (Norwegian Mother and Child Cohort Study), Norway, 1999–2009
**Table S4.** Relative risks of Congenital Heart Defect* (Overall), Severe Heart Defect^†^, Conotruncal Defect^‡^, and Septal Defect^§^ by Maternal Intake of Folic Acid Supplements, Adjusting for Covariates Using 3 Models, Combining 94 228 Births^∥^ in the DNBC (Danish National Birth Cohort), Denmark, 1996–2003, and 102 985 Births^∥^ in MoBa (Norwegian Mother and Child Cohort Study), Norway, 1999–2009
**Table S5.** Relative Risk of Congenital Heart Defects* (Overall), by Maternal Intake of Folic Acid Supplements in the Periconceptional Period, in Sensitivity Analyses With Restrictions to Live Births, Singleton Births, Mother Without Heart Defect, or Planned Pregnancies, Combining 94 228 Births in the DNBC (Danish National Birth Cohort), Denmark, 1996–2003, and 102 985 Births in MoBa (Norwegian Mother and Child Cohort Study), Norway, 1999–2009

## References

[1] Jenkins KJ , Correa A , Feinstein JA , Botto LD , Britt AE , Daniels SR , Elixson M , Warnes CA , Webb CL . Non‐inherited risk factors and congenital cardiovascular defects: current knowlegde: a scientific statement from the American Heart Association Council on Cardiocascular Disease in the Young: endorsed by the American Academy of Pediatrics. Circulation. 2007;115:2995–3014. 17519397 10.1161/CIRCULATIONAHA.106.183216

[2] Pierpont ME , Basson CT , Benson DW , Gelb BD , Giglia TM , Goldmuntz E , McGee G , Sable CA , Srivastava D , Webb CL . Genetic basis for congenital heart defects: current knowledge: a scientific statement from the American Heart Association Congenital Cardiac Defects Committee, Council on Cardiovascular Disease in the Young: endorsed by the American Academy of Pediatrics. Circulation. 2007;115:3015–3038. 17519398 10.1161/CIRCULATIONAHA.106.183056

[3] Fahed AC , Gelb BD , Seidman JG , Seidman CE . Genetics of congenital heart disease: the glass half empty. Circ Res. 2013;112:707–720. 23410880 10.1161/CIRCRESAHA.112.300853 PMC3827691

[4] Øyen N , Poulsen G , Boyd HA , Wohlfahrt J , Jensen PKA , Melbye M . Recurrence of congenital heart defects in families. Circulation. 2009;120:295–301. 19597048 10.1161/CIRCULATIONAHA.109.857987

[5] Group MVSR . Prevention of neural tube defects. Results of the Medical Research Council Vitamin Study. Lancet. 1991;338:131–137. 1677062

[6] Czeizel A , Dudas I . Prevention of the first occurence of neural tube defects by periconceptional vitamin supplementation. N Engl J Med. 1992;327:1832–1835. 1307234 10.1056/NEJM199212243272602

[7] Shaw GM , O'Malley CD , Wasserman CR , Tolarova MM , Lammer EJ . Maternal periconeptional use of multivitamins and reduced risk for conotruncal heart defects and limb deficiencies among offspring. Am J Med Genet. 1995;59:536–545. 8585581 10.1002/ajmg.1320590428

[8] van Beynum IM , Kapusta L , Bakker MK , den Heijer M , Blom HJ , de Walle HE . Protective effect of periconceptional folic acid supplements on the risk of congenital heart defects: a registry‐based case‐control study in the northern Netherlands. Eur Heart J. 2010;31:464–471. 19952004 10.1093/eurheartj/ehp479

[9] Tang LS , Wlodarcyk BJ , Santillano DR , Miranda RC , Finell RH . Developmental consequences of abnormal folate transport during murin heart morphogenesis. Birth Defects Res A Clin Mol Teratol. 2004;70:449–458. 15259034 10.1002/bdra.20043

[10] Van Beynum IM , Kapusta L , den Heijer M , Vermeulen SH , Kouwenberg M , Daniels O , Blom HJ . Maternal MTHFR 677C>T is a risk factor for congenital heart defects: effect modificaition by periconceptional folate supplementation. Eur Heart J. 2009;27:981–987. 10.1093/eurheartj/ehi815 16524890

[11] Czeizel AE . Prevention of congenital abnormalities by periconceptional multivitamin supplementation. BMJ. 1993;306:1645–1648. 8324432 10.1136/bmj.306.6893.1645 PMC1678103

[12] Li X , Li S , Mu D , Liu Z , Li Y , Lin Y , Chen X , You F , Li N , Deng K , Deng Y , Wang Y , Zhu J . The association between periconceptional folic acid supplementation and congenital heart defects: a case‐control study in China. Prev Med. 2013;56:385–389. 23480969 10.1016/j.ypmed.2013.02.019

[13] Obermann‐Borst SA , Isaacs A , Younes Z , van Schaik RH , van der Heiden IP , van Duyn CM , Steegers EA , Steegers‐Theunissen RP . General maternal medication use, folic acid, the MDR1 C3435T polymorphism, and the risk of a child with a congenital heart defect. Am J Obstet Gynecol. 2011;204:236.e1–8. 21183151 10.1016/j.ajog.2010.10.911

[14] Bower C , Miller M , Payne J , Serna P . Folate intake and the primary prevention of non‐neural birth defects. Aust N Z J Public Health. 2006;30:258–261. 16800203 10.1111/j.1467-842x.2006.tb00867.x

[15] Werler MM , Hayes C , Louik C , Shapiro S , Mitchell AA . Multivitamin supplementation and risk of birth defects. Am J Epidemiol. 1999;150:675–682. 10512421 10.1093/oxfordjournals.aje.a010070

[16] Scanlon KS , Ferencz C , Loffredo CA , Wilson PD , Correa‐Villasenor A , Khoury MJ , Willett WC . Preconceptional folate intak and malformations of the cardiac outflow tract. Baltimore‐Washington Infant Study Group. Epidemiology. 1998;9:95–98. 9430276

[17] Leirgul E , Gildestad T , Nilsen RM , Fomina T , Brodwall K , Greve G , Vollset SE , Holmstrøm H , Tell GS , Øyen N . Periconceptional folic acid supplementation and infant risk of congenital heart defects in Norway 1999–2009. Paediatr Perinat Epidemiol. 2015;29:391–400. 26212116 10.1111/ppe.12212

[18] Bell KN , Oakley GP . Update on prevention of folic acid‐preventable spina bifida and anencephaly. Birth Defects Res A Clin Mol Teratol. 2009;85:102–107. 19067404 10.1002/bdra.20504

[19] Botto LD , Olney RS , Erickson JD . Vitamin supplements and the risk for congenital anomalies other than neural tube defects. Am J Med Genet C Semin Med Genet. 2004;125C:12–21. 14755429 10.1002/ajmg.c.30004

[20] Wen SW , Liu S , Joseph KS , Rouleau J , Allen A . Pattern of infant mortality caused by major congenital anomalies. Teratology. 2000;61:342–346. 10777829 10.1002/(SICI)1096-9926(200005)61:5<342::AID-TERA5>3.0.CO;2-7

[21] Olsen J , Melbye M , Olsen SF , Sørensen TI , Aaby P , Andersen AM , Taxbol D , Hansen KD , Juhl M , Schow TB , Sørensen HT , Andresen J , Mortensen EL , Olesen AW , Søndergaard C . The Danish National Birth Cohort—its background, structure and aim. Scand J Public Health. 2001;29:300–307. 11775787 10.1177/14034948010290040201

[22] Magnus P , Birke C , Vejrup K , Haugan A , Alsaker E , Daltveit AK , Handal M , Haugen M , Høiseth G , Knudsen GP , Paltiel L , Schreuder P , Tambs K , Vold L , Stoltenberg C . Cohort profile update: the Norwegian Mother and Child Cohort Study (MoBa). Int J Epidemiol. 2016;45:382–388. 27063603 10.1093/ije/dyw029

[23] Øyen N , Poulsen G , Boyd HA , Wohlfahrt J , Jensen PKA , Melbye M . National time trends in congenital heart defects, Denmark, 1977–2005. Am Heart J. 2009;157:467–473. 19249416 10.1016/j.ahj.2008.10.017

[24] Agergaard P , Hebert A , Bjerre J , Sørensen KM , Olesen C , Østergaard JR . Children diagnosed with congenital cardiac malformations at the national university departments of pediatric cardiology: positive predictive values of data in the Danish National Patient Registry. Clin Epidemiol. 2011;3:61–66. 21386975 10.2147/CLEP.S15627 PMC3046186

[25] Helweg‐Larsen K . The Danish register of causes of death. Scand J Public Health. 2011;39:26–29. 10.1177/1403494811399958 21775346

[26] Leirgul E , Fomina T , Brodwall K , Greve G , Holmstrøm H , Vollset SE , Tell GS , Øyen N . Birth prevalence of congenital heart defects in Norway 1994–2009—a nationwide study. Am Heart J. 2014;168:956–964. 25458661 10.1016/j.ahj.2014.07.030

[27] Strøm M , Granstrøm C , Lyall K , Ascherio A , Olsen SF . Research Letter: folic acid supplementation and intake of folate in pregnancy in relation to offspring risk of autism spectrum disorder. Psychol Med. 2018;48:1048–1054. 28946926 10.1017/S0033291717002410

[28] Nilsen RM , Vollset SE , Gjessing H , Magnus P , Meltzer HM , Haugen M , Ueland PM . Patterns and predictors of folic acid supplement use among pregnant women: the Norwegian Mother and Child Cohort Study. Am J Clin Nutr. 2006;84:1134–1141. 17093167 10.1093/ajcn/84.5.1134

[29] Olsen SF , Knudsen VK . Folic acid for the prevention of neural tube defects: the Danish experience. Food Nutr Bull. 2008;29:S205–S209. 18709894 10.1177/15648265080292S124

[30] Olsen SF , Mikkelsen TB , Knudsen VK , Orozova‐Bekkevold I , Halldorsson TI , Strøm M , Østerdal ML . Data collected on maternal dietetary exposures in the Danish National Birth Cohort. Paediatr Perinat Epidemiol. 2007;21:76–86. 17239183 10.1111/j.1365-3016.2007.00777.x

[31] Mikkelsen TB , Osler M , Olsen SF . Validity of protein, retinol, folic acid and n‐3 fatty acid intakes estimated from the food‐frequency questionnaire used in the Danish National Birth Cohort. Public Health Nutr. 2006;9:771–778. 16925883 10.1079/phn2005883

[32] Roth C , Bjørke‐Monsen AL , Reichborn‐Kjennerud T , Nilsen RM , Smith GD , Stoltenberg C , Suren P , Susser E , Ueland PM , Vollset SE , Magnus P . Use of folic acid supplements in early pregnancy in relation to maternal plasma levels in week 18 of pregnancy. Mol Nutr Food Res. 2013;57:653–660. 23065724 10.1002/mnfr.201200116 PMC3882014

[33] Kirby ML . Cardiac Development. Oxford, United Kingdom: Oxford University Press; 2009.

[34] Roth C , Magnus P , Schjolberg S , Stoltenberg C , Suren P , McKeague IW , Davey Smith G , Reichborn‐Kjennerud T , Susser E . Folic acid supplements in pregnancy and severe language delay in children. JAMA. 2011;306:1566–1573. 21990300 10.1001/jama.2011.1433 PMC3780384

[35] Suren P , Roth C , Bresnahan M , Haugen M , Hornig M , Hirtz D , Lie KK , Lipkin WI , Magnus P , Reichborn‐Kjennerud T , Schjolberg S , Davey Smith G , Øyen AS , Susser E , Stoltenberg C . Association between maternal use of folic acid supplements and risk of autism spectrum disorders in children. JAMA. 2013;309:570–577. 23403681 10.1001/jama.2012.155925 PMC3908544

[36] Botto LD , Lin AE , Riehle‐Colarusso T , Malik S , Correa A ; National Birth Defects Prevention Study . Seeking causes: classifying and evaluating congenital heart defects in etiologic studies. Birth Defects Res A Clin Mol Teratol. 2007;79:714–727. 17729292 10.1002/bdra.20403

[37] Olsen SF , Michaelsen KF , Rasmussen LB , Knudsen VK . Folsyre til kvinder, der planlægger graviditet—kun få følger anbefalingen! Copenhagen: Ernæringsrådet/Boje & Mobeck as; 2003.

[38] Ionescu‐Ittu R , Marelli AJ , Mackie AS , Pilote L . Prevalence of severe congenital heart disease after folic acid fortification of grain products: time trend analysis in Quebec, Canada. BMJ. 2009;338:b1673. 19436079 10.1136/bmj.b1673 PMC2682153

[39] Liu S , Joseph KS , Luo W , Leon JA , Lisonkova S , Van den Hof M , Evans J , Lim K , Little J , Sauve R , Kramer MS ; Canadian Perinatal Surveillance System (Public Health Agency of Canada) . Effect of folic acid food fortification in Canada on congenital heart disease subtypes. Circulation. 2016;134:647–655. 27572879 10.1161/CIRCULATIONAHA.116.022126 PMC4998126

[40] Correa A , Gilboa SM , Botto LD , Moore CA , Hobbs CA , Cleves MA , Riehle‐Colarusso TJ , Waller DK , Reece EA ; National Birth Defects Prevention Study . Lack of periconceptional vitamins or supplements that contain folic acid and diabetes mellitus‐associated birth defects. Am J Obstet Gynecol. 2012;206:218. 22284962 10.1016/j.ajog.2011.12.018 PMC4915337

[41] Malik S , Cleves MA , Honein MA , Romitti PA , Botto LD , Yang S , Hobbs CA ; National Birth Defects Prevention S . Maternal smoking and congenital heart defects. Pediatrics. 2008;121:e810–e816. 18381510 10.1542/peds.2007-1519

[42] Shaw GM , Carmichael SL , Yang W , Lammer EJ . Periconceptional nutrient intakes and risks of conotruncal heart defects. Birth Defects Res A Clin Mol Teratol. 2010;88:144–151. 20063270 10.1002/bdra.20648 PMC2849796

[43] Csaky‐Szunyogh M , Vereczkey A , Kosa Z , Gerencser B , Czeizel AE . Risk and protective factors in the origin of conotruncal defects of heart—a population‐based case‐control study. Am J Med Genet A. 2013;161A:2444–2452. 23950097 10.1002/ajmg.a.36118

[44] Khoshnood B , Loane M , Garne E , Addor MC , Arriola L , Bakker M , Barisic I , Bianca S , Boyd P , Calzolari E , Doray B , Draper E , Gatt M , Haeusler M , Melve KK , Latos‐Bielenska A , McDonnell B , Mullaney C , Nelen V , O'Mahony M , Pierini A , Queisser‐Luft A , Randrianaivo H , Rankin J , Rissmann A , Salvador J , Tucker D , Verellen‐Dumoulin C , Wellesley D , Zymak‐Zakutnya N , Dolk H . Recent decrease in the prevalence of congenital heart defects in Europe. J Pediatr. 2013;162:108–113.e2. 22835879 10.1016/j.jpeds.2012.06.035

[45] Mamasoula C , Prentice RR , Pierscionek T , Pangilinan F , Mills JL , Druschel C , Pass K , Russell MW , Hall D , Töpf A , Brown DL , Zelenika D , Bentham J , Cosgrove C , Bhattacharya S , Riveron JG , Setchfield K , Brook JD , Bu'Lock FA , Thornborough C , Rahman TJ , Doza JP , Tan HL , O'Sullivan J , Stuart AG , Blue G , Winlaw D , Postma AV , Mulder BJM , Zwinderman AH , van Engelen K , Moorman AFM , Rauch A , Gewillig M , Breckpot J , Devriendt K , Lathrop GM , Farrall M , Goodship JA , Cordell HJ , Brody LC , Keavney BD . Association between C677T polymorphism of methylene tetrahydrofolate reductase and congenital heart disease: meta‐analysis of 7697 cases and 13 125 controls. Circ Cardiovasc Genet. 2013;6:347–353. 23876493 10.1161/CIRCGENETICS.113.000191 PMC3855044

[46] Mortensen JH , Øyen N , Fomina T , Melbye M , Tretli S , Vollset SE , Bjørge T . Supplemental folic acid in pregnancy and maternal cancer risk. Cancer Epidemiol. 2015;39:805–811. 26569032 10.1016/j.canep.2015.10.009

[47] Mortensen JH , Øyen N , Fomina T , Melbye M , Tretli S , Vollset SE , Bjørge T . Supplemental folic acid in pregnancy and childhood cancer risk. Br J Cancer. 2016;114:71–75. 26757423 10.1038/bjc.2015.446 PMC4716548

[48] Brantsaeter AL , Haugen M , Alexander J , Meltzer HM . Validity of a new food frequency questionnaire for pregnant women in the Norwegian Mother and Child Cohort Study (MoBa). Matern Child Nutr. 2008;4:28–43. 18171405 10.1111/j.1740-8709.2007.00103.x PMC6860878

[49] Bjørke‐Monsen AL , Roth C , Magnus P , Midttun O , Nilsen RM , Reichborn‐Kjennerud T , Stoltenberg C , Susser E , Vollset SE , Ueland PM . Maternal B vitamin status in pregnancy week 18 according to reported use of folic acid supplements. Mol Nutr Food Res. 2013;57:645–652. 23001761 10.1002/mnfr.201200114 PMC3774931

[50] Tollånes MC , Strandberg‐Larsen K , Forthun I , Petersen TG , Moster D , Andersen AM , Stoltenberg C , Olsen J , Wilcox AJ . Cohort profile: cerebral palsy in the Norwegian and Danish birth cohorts (MOBAND‐CP). BMJ Open. 2016;6:e012777. 10.1136/bmjopen-2016-012777 PMC5020679 27591025

