# Supplementary material for: Association Between Maternal Folic Acid Supplementation and Congenital Heart Defects in Offspring in Birth Cohorts From Denmark and Norway
Source: J Am Heart Assoc. 2019 Mar 12;8(6):e011615. doi: 10.1161/JAHA.118.011615 (PMC6475034; doi:10.1161/JAHA.118.011615)
Supplement: Supplementary file 1 — Data S1. A detailed discussion on previous findings from studies of the association between folic acid and congenital heart defects. Table S1. The Classification System for Congenital Heart Defect Phenotypes Table S2. Relative Risk of Congenital Heart Defect* (Overall), Severe Heart Defect†, Conotruncal Defect‡, and Septal Defect§ by Maternal Intake of Folic Acid Supplements in the Periconceptional Period Among 94 228 Births the DNBC (Danish National Birth Cohort), Denmark, 1996–2003 Table S3. Relative Risk of congenital Heart Defect* (Overall), Severe Heart Defect†, Conotruncal Defect‡, and Septal Defect§ by Maternal Intake of Folic Acid Supplements in the Periconceptional Period Among 102 985 Births in MoBa (Norwegian Mother and Child Cohort Study), Norway, 1999–2009 Table S4. Relative risks of Congenital Heart Defect* (Overall), Severe Heart Defect†, Conotruncal Defect‡, and Septal Defect§ by Maternal Intake of Folic Acid Supplements, Adjusting for Covariates Using 3 Models, Combining 94 228 Births∥ in the DNBC (Danish National Birth Cohort), Denmark, 1996–2003, and 102 985 Births∥ in MoBa (Norwegian Mother and Child Cohort Study), Norway, 1999–2009 Table S5. Relative Risk of Congenital Heart Defects* (Overall), by Maternal Intake of Folic Acid Supplements in the Periconceptional Period, in Sensitivity Analyses With Restrictions to Live Births, Singleton Births, Mother Without Heart Defect, or Planned Pregnancies, Combining 94 228 Births in the DNBC (Danish National Birth Cohort), Denmark, 1996–2003, and 102 985 Births in MoBa (Norwegian Mother and Child Cohort Study), Norway, 1999–2009 [file JAH3-8-e011615-s001.pdf]

SUPPLEMENTAL MATERIAL

Data S1.

A detailed discussion on previous findings from studies of the association between folic acid and congenital heart defects

From the United States, there are six case-control studies worth mentioning. From California, 1987-1998, mothers of 207 children with conotruncal heart defects and 481 randomly selected infants without malformations were telephone interviewed.[1] There was a reduced risk of conotruncal heart defects in children of mothers who had taken multivitamins or folic acid fortified cereals (OR 0.70 95% CI 0.46-1.1), but with a non-significant estimate. In a later study from California, 1999-2004, no preventive effect of multivitamins with folic acid on conotruncal defects was found, when comparing 186 infants with transposition of the great arteries or Tetralogy of Fallot (ToF) to 426 live controls.[2] In the Slone Epidemiology Unit Birth Defects Study, 1993-1996, 101 infants with conotruncal defects, 86 ventricular septal defect (VSD), and 521 controls were recruited from hospitals in Boston, Philadelphia, and Toronto, Canada. No significant association were found between prenatal vitamins containing folic acid, and the types of heart defect.[3] In the Baltimore-Washington Infant Study, 1987-1989, 126 non-syndromic outflow tract defects (53 transposition of the great arteries (TGA) and 73 other, e.g. ToF, double outlet right ventricle (DORV), truncus arteriosus (TA), supracristal VSD) were compared with 679 controls; the authors found no preventive effect of folic acid supplementations before pregnancy, a finding also supported by null findings for folic acid intake in the 5 weeks pregestational period, and for the lowest quartile of total folate intake.[4] In a study from the US National Birth Defects Prevention Study, 1997-2002, with the intention to investigate the association between maternal smoking and offspring heart defect,[5] the authors also reported the potential confounder maternal folic acid intake in the periconceptional period one month

before and 2 months after conception. By our calculation of the authors' numbers (2284 exposed heart defect out of 3,067 all heart defects; 2,935 non-exposed controls out of 3947 controls), the unadjusted OR was 1.01 (95% CI 0.90-1.12), i.e. the association between folic acid and heart defect risk was null. In another report from the National Birth Defects Prevention Study, 1997-2004 (which might overlap with the study above), from Arkansas, California, Georgia (Atlanta), Iowa, Massachusetts, New Jersey (through 2002), New York, North Carolina (beginning 2003), Texas, Utah (from 2003 onwards) on the joint effect of maternal diabetes and folic acid containing multivitamins on the risk of different types of heart defect in a multi-center study,[6] we calculated an unadjusted OR of 0.95 (95% CI 0.85-1.06) of any heart defect for maternal use of multivitamins with folic acid (5,205 exposed with heart defects out of 5,979 individuals with heart defects; 4,737 exposed controls out of 5,408 controls).

The Hungarian randomized controlled trial of neural tube defects,[7] containing other defects as well, were extended with 8 months follow-up,[8] and reported a reduced risk of heart defect, but the difference was non-significant comparing the group receiving multivitamin supplements containing high dose of 0.8 mg folic acid with the group receiving trace element supplements other than folic acid. The numbers were small, with only 10 exposed and 17 unexposed heart defect cases. In a later follow-up, but in case-control design, 598 children with heart defect born in Hungary 1980-1996 were compared to 902 matched controls,[9] The authors reported a reduced risk of conotruncal heart defect in the group exposed to a very high dose of folic acid, on average 5.6 mg/d.

In Western Australia, 1997-1998, with no folate fortification of staple food, there was no association between low dose folic acid (0.2 mg or more) and offspring heart defect risk comparing several birth defect outcomes, including 151 heart defects, to 578 live controls without birth defects, syndromes or chromosomal aberrations.[10]

Two recent Dutch case-control studies report from different areas. From the Northern Netherlands, 1996-2005, including 611 children with heart defect identified within a birth defect registry, and two control groups; 2,401 supposedly non-folate related birth defects, and 3,343 births of women participating in previous cross-sectional studies, reported an adjusted ORs of 0.82 (95% CI 0.68-0.98) and 0.74 (95% CI 0.62-0.88) of heart defect among offspring of women using periconceptional supplements with 0.4 mg/d folic acid comparing birth defect controls and live controls, respectively.[11] However, from the Western Netherlands, 2003 onwards, the authors report a non-significant reduction of heart defect for folic acid intake of 0.4 mg or more (p=0.16), by our calculation, OR 0.79 (95% CI 0.57-1.10) when comparing 283 heart defect (ToF, TGA, atrioventricular septal defect (AVSD), Coarctation of aorta, Aortic valve stenosis, pulmonary valve stenosis, Hypoplastic left heart syndrome) to 308 controls.[12]

From China, a hospital-based case-control study with 358 prenatally identified heart defect and 422 controls recruited consecutively among women receiving prenatal investigation in the provinces of Guangdong, Hubei, Fujian, and Shanxi, folic acid supplements reduced heart defect risk by 65%.[13] However, there may be limitations of the study, not discussed by the authors, such as design, selection into the study, and classification of exposure.

In the recent study of around 520,000 births registered in the Medical Birth Registry of Norway, 1999-2009, with pre-birth registered use of folic acid and multivitamin supplements before pregnancy and during pregnancy,[14] the authors identified 1,153 births with severe type of heart defect (heterotaxia, conotruncal defects, AVSD, anomalous pulmonary venous return (APVR), left or right ventricle outlet tract obstructions (LVOTO/RVOTO), other specified defects) and 3,280 with isolated septal defects (VSD, atrial septal defect (ASD), VSD+ASD). There was no significant association between intake of folic acid supplements (containing 0.4 mg folic acid) and severe heart defect, but a surprising 20% increase of septal defects.

Table S1. The classification system for congenital heart defect phenotypes.

Main groups	Detailed cardiac phenotypes
Heterotaxia	Situs inversus<br>Isomerism<br>Dextrocardia or levocardia with other heart defect
Conotruncal defects	Truncus arteriosus<br>Transposition of the great arteries (TGA)<br>Tetralogy of Fallot (ToF)<br>Pulmonary atresia with ventricular septal defect (ToF type)<br>Double outlet right ventricle (DORV)<br>Conoventricular septal defect<br>Interrupted aortic arch type B or C<br>Supravalvular aortic stenosis<br>Aortopulmonary window
Atrioventricular septal defects	Atrioventricular septal defects
Anomalous pulmonary venous return (APVR)	Total anomalous pulmonary venous return<br>Partial anomalous pulmonary venous return
Left Ventricular Outflow Tract Obstructions (LVOTO)	Hypoplastic left heart syndrome (HLHS)<br>Mitral valve stenosis<br>Coarctation of the aorta (CoA)<br>Interrupted aortic arch type A<br>Valvular aortic stenosis
Right Ventricular Outflow Tract Obstructions (RVOTO)	Tricuspid atresia / stenosis<br>Hypoplastic right heart syndrome (HRHS)<br>Ebstein anomaly<br>Valvular pulmonary atresia (not ToF anatomy)<br>Arterial pulmonary atresia<br>Valvular pulmonary stenosis
Septal defects	Atrial septal defects (ASD)<br>Ventricular septal defects (VSD)<br>ASD + VSD<br>Otherwise specified or not specified septal defects
Other complex cardiac defects	Congenitally corrected transposition of the great arteries (ccTGA)<br>Single ventricle (non-HLHS, non-HRHS)<br>Double inlet left ventricle (DOLV)<br>Absent PV
Other cardiac defects	Infundibular pulmonary stenosis<br>Pulmonary insufficiency<br>Subaortic stenosis<br>Aortic insufficiency<br>Mitral insufficiency<br>Pulmonary arterial stenosis<br>Cor triatriatum<br>Coronary malformations<br>Other specified malformation of the heart<br>Unspecified malformations of the heart, great arteries, great veins
Isolated Patent ductus arteriosus (PDA)	Isolated patent ductus arteriosus (PDA)

Severe heart defects: Heterotaxia, Conotruncal defects, Atrioventricular septal defect, APVR, LVOTO, RVOTO (except valvular pulmonary stenosis), Other complex defects.

Table S2. Relative risk (RR) of congenital heart defect* (overall), severe heart defect†, conotruncal defect‡, and septal defect§ by maternal intake of folic acid supplements in the periconceptional period among 94,228 births The Danish National Birth Cohort (DNBC), Denmark, 1996-2003.

	Total no. of births (%)‖	Any congenital heart defect* N=995				Severe heart defect† N=303				Conotruncal heart defect‡ N=105				Septal defect§ N=445			
	94,228	N	No. per 10,000	Crude RR 95% CI	Adjusted RR# 95% CI	N	No. per 10,000	Crude RR 95% CI	Adjusted RR# 95% CI	N	No. per 10,000	Crude RR 95% CI	Adjusted RR# 95% CI	N	No. per 10,000	Crude RR 95% CI	Adjusted RR# 95% CI
Supplement use (4 weeks before to 8 weeks after conception)																	
None	31,322 (33.2)	311	99	1	1	100	32	1	1	36	11	1	1	134	43	1	1
Other supplements, no folic acid	3,453 (3.7)	43	125	1.25 0.91-1.72	1.35 0.97-1.87	9	26	0.82 0.41-1.62	0.89 0.45-1.76	2	6	0.50 0.12-2.09	0.53 0.13-2.22	26	75	1.76 1.16-2.67	1.95 1.27-3.01
Folic acid only	3,889 (4.1)	36	93	0.93 0.66-1.31	0.95 0.67-1.35	8	21	0.65 0.31-1.32	0.63 0.31-1.31	4	10	0.90 0.32-2.52	0.93 0.33-2.64	15	39	0.90 0.53-1.54	0.94 0.55-1.61
Folic acid plus other supplementation	55,564 (59.0)	605	109	1.10 0.96-1.26	1.12 0.97-1.30	186	33	1.05 0.82-1.34	1.01 0.79-1.31	63	11	0.99 0.66-1.49	0.90 0.58-1.38	270	49	1.14 0.92-1.40	1.14 0.92-1.42
Initiation of folic acid** (4 weeks before to 11 weeks after conception)																	
None	32,252 (34.9)	323	100	1	1	101	31	1	1	38	12	1	1	142	44	1	1
Week -4 to -1	32,917 (34.2)	360	109	1.09 0.94-1.27	1.13 0.96-1.32	106	32	1.03 0.78-1.35	1.01 0.75-1.36	38	12	0.98 0.63-1.54	0.89 0.56-1.44	161	49	1.11 0.89-1.39	1.13 0.89-1.43
Week 0 to 4	10,785 (11.5)	107	99	0.99 0.80-1.23	0.98 0.78-1.24	31	29	0.92 0.61-1.37	0.88 0.57-1.36	10	9	0.79 0.39-1.58	0.63 0.29-1.37	47	44	0.99 0.71-1.38	0.99 0.70-1.39
Week 5 to 8	15,751 (16.7)	174	110	1.10 0.92-1.32	1.08 0.89-1.32	57	36	1.16 0.84-1.60	1.12 0.78-1.61	19	12	1.02 0.59-1.78	0.99 0.55-1.77	77	49	1.11 0.84-1.47	1.00 0.74-1.35
Week 9 to 11	2,523 (2.7)	31	123	1.23 0.85-1.77	1.16 0.78-1.73	8	32	1.01 0.50-2.15	0.76 0.31-2.08	0				18	71	1.62 0.99-2.64	1.45 0.85-2.47

*†‡§ Congenital heart defects: see definitions in Table 4.

‖ The number of events includes the entire cohort, whereas in the adjusted analyses, individuals with missing values for covariates were excluded (total births n=7,556; any heart defect n=85; severe defect n=22, conotruncal defects n=10; septal defects n=36).

Relative risk (RR) with 95% confidence interval (CI) comparing supplement use with no use (reference) 4 weeks before to 8 weeks after conception (upper panel) or comparing initiation of folic acid supplements with no use/non-folic acid supplements (reference) 4 weeks before to 12 weeks after conception (lower panel). RRs adjusted for year of birth (1996-97, 1998, 1999, 2000, 2001, 2002-03), maternal age (≤24, 25-29, 30-34, ≥35 years), birth order (1, 2, 3+), maternal body mass index (<20, 20-24, 25-29, 30-34, ≥35), maternal heart defect (yes/no), maternal epilepsy before pregnancy (yes/no). Categories for adjustment variables were combined for severe and conotruncal defects.

** The four exposure categories in supplement use collapsed into no/yes; “no” is no folic acid use (no supplements and other supplements, no folic acid), and “yes” is folic acid use (folic acid only and folic acid plus other supplementation).

Table S3. Relative risk (RR) of congenital heart defect* (overall), severe heart defect†, conotruncal defect‡, and septal defect§ by maternal intake of folic acid supplements in the periconceptional period among 102,985 births in the Norwegian Mother and Child Cohort Study (MoBa), Norway, 1999-2009.

	Total no. of births (%)‖	Any congenital heart defect* N=1,252				Severe heart defect† N=280				Conotruncal heart defect‡ N=96				Septal defect§ N=746			
	102,985	N	No. per 10,000	Crude RR 95% CI	Adjusted RR# 95% CI	N	No. per 10,000	Crude RR 95% CI	Adjusted RR# 95% CI	N	No. per 10,000	Crude RR 95% CI	Adjusted RR# 95% CI	N	No. per 10,000	Crude RR 95% CI	Adjusted RR# 95% CI
Supplement use (4 weeks before to 8 weeks after conception)																	
None	25,229 (24.5)	312	124	1	1	70	28	1	1	23	9	1	1	184	73	1	1
Other supplements, no folic acid	6,431 (6.2)	65	101	0.82 0.63-1.07	0.83 0.64-1.09	16	25	0.90 0.52-1.54	0.97 0.56-1.67	6	9	1.02 0.42-2.51	1.08 0.43-2.67	36	56	0.77 0.54-1.10	0.77 0.54-1.11
Folic acid only	19,555 (19.0)	248	127	1.03 0.87-1.21	1.07 0.90-1.27	62	32	1.14 0.81-1.61	1.25 0.87-1.79	19	10	1.07 0.58-1.96	1.07 0.56-2.03	142	73	1.00 0.80-1.24	1.00 0.80-1.25
Folic acid plus other supplementation	51,770 (50.3)	627	121	0.98 0.86-1.12	1.02 0.88-1.18	132	25	0.92 0.69-1.23	1.03 0.75-1.42	48	9	1.02 0.62-1.67	1.00 0.58-1.73	384	74	1.02 0.85-1.21	1.00 0.83-1.21
Initiation of folic acid** (4 weeks before to 12 weeks after conception)																	
None	25,084 (24.3)	304	121	1	1	66	26	1	1	23	10	1	1	178	71	1	1
Week -4 to -1	32,827 (31.9)	417	127	1.05 0.91-1.21	1.09 0.93-1.28	95	29	1.10 0.80-1.50	1.25 0.88-1.76	32	10	1.06 0.62-1.82	1.07 0.59-1.93	248	76	1.06 0.88-1.29	1.04 0.85-1.28
Week 0 to 5	19,471 (18.9)	254	130	1.08 0.91-1.27	1.13 0.95-1.35	54	28	1.05 0.74-1.51	1.19 0.82-1.73	21	11	1.18 0.65-2.13	1.21 0.65-2.27	157	81	1.14 0.92-1.41	1.13 0.91-1.42
Week 5 to 8	19,027 (18.5)	204	107	0.89 0.74-1.06	0.91 0.76-1.09	45	24	0.90 0.62-1.31	0.91 0.61-1.36	14	7	0.80 0.41-1.56	0.75 0.37-1.51	121	64	0.90 0.71-1.13	0.88 0.70-1-12
Week 9 to 12	6,576 (6.4)	73	111	0.92 0.71-1.18	0.90 0.70-1.17	20	30	1.15 0.70-1.90	1.08 0.64-1.83	6	9	0.99 0.40-2.44	1.04 0.42-2.59	42	64	0.90 0.64-1.26	0.88 0.63-1.24

*†‡§ Congenital heart defects: see definitions in Table 4

‖ The number of events includes the entire cohort, whereas in the adjusted analyses, individuals with missing values for covariates were excluded (total births n=2,796; any heart defect n=32; severe defect n=10, conotruncal defects n=4; septal defects n=16).

Relative risk (RR) with 95% confidence interval (CI) comparing supplement use with no use (reference) 4 weeks before to 8 weeks after conception (upper panel) or comparing initiation of folic acid supplements with no use/non-folic acid supplements (reference) 4 weeks before to 12 weeks after conception (lower panel). RRs adjusted for year of birth (1999-2000, 2001, 2002, 2003, 2004, 2005, 2006, 2007, 2008-09), maternal age (years ≤24, 25-29, 30-34, ≥35), birth order (1, 2, 3+), maternal body mass index (<20, 20-24, 25-29, 30-34, ≥35), maternal heart defect (yes/no), maternal epilepsy before pregnancy (yes/no). Categories for adjustment variables combined for severe and conotruncal defects.

** The four exposure categories in supplement use collapsed into no/yes; “no” is no folic acid use (no supplements and other supplements, no folic acid), and “yes” is folic acid use (folic acid only and folic acid plus other supplementation).

Table S4. Relative risks (RR) of congenital heart defect* (overall), severe heart defect†, conotruncal defect‡, and septal defect§ by maternal intake of folic acid supplements, adjusting for covariates using three models, combining 94,228 births‖ in The Danish National Birth Cohort (DNBC), Denmark, 1996-2003, and 102,985 births‖ in The Norwegian Mother and Child Cohort Study (MoBa), Norway, 1999-2009.

	Any congenital heart defect*			Severe heart defect†			Conotruncal defect‡			Septal defect§		
	Adjusted RR#<br>95% CI			Adjusted RR#<br>95% CI			Adjusted RR#<br>95% CI			Adjusted RR#<br>95% CI		
	Model 1<br>n=2,130	Model 2<br>n=2,037	Model 3<br>N=1,916	Model 1<br>n=551	Model 2<br>n=524	Model 3<br>n=497	Model 1<br>n=187	Model 2<br>n=180	Model 3<br>n=170	Model 1<br>n=1,139	Model 2<br>n=1,090	Model 3<br>n=1,025
Supplement use<br>(4 weeks before<br>to 8 weeks after conception)												
None	1	1	1	1	1	1	1	1	1	1	1	1
Other supplements,<br>no folic acid	0.99<br>0.80,1.22	1.01<br>0.82,1.24	1.10<br>0.89,1.38	0.92<br>0.61,1.41	0.89<br>0.57,1.38	1.01<br>0.64,1.58	0.84<br>0.40,1.77	0.79<br>0.36,1.75	0.76<br>0.32,1.78	1.06<br>0.80,1.39	1.07<br>0.81,1.42	1.20<br>0.89,1.60
Folic acid only	1.08<br>0.93,1.25	1.12<br>0.97,1.30	1.15<br>0.98,1.35	1.10<br>0.82,1.48	1.09<br>0.81,1.48	1.21<br>0.88,1.66	0.98<br>0.59,1.64	1.00<br>0.59,1.69	1.13<br>0.66,1.94	1.03<br>0.84,1.26	1.05<br>0.86,1.29	1.07<br>0.86,1.33
Folic acid plus other supplementation	1.07<br>0.97,1.19	1.06<br>0.96,1.18	1.10<br>0.99,1.23	1.02<br>0.84,1.25	0.99<br>0.81,1.22	1.02<br>0.83,1.26	0.93<br>0.66,1.31	0.88<br>0.62,1.25	0.93<br>0.65,1.33	1.06<br>0.92,1.22	1.07<br>0.93,1.24	1.11<br>0.95,1.29
Initiation of folic acid**<br>(4 weeks before to 12 weeks after conception)												
None	1	1	1	1	1	1	1	1	1	1	1	1
Week -4 to -1	1.11<br>1.00,1.25	1.13<br>1.01,1.27	1.15<br>1.02,1.30	1.08<br>0.87,1.34	1.06<br>0.85,1.32	1.11<br>0.88,1.40	0.95<br>0.66,1.38	0.93<br>0.64,1.37	0.99<br>0.67,1.46	1.08<br>0.92,1.26	1.10<br>0.94,1.30	1.13<br>0.96,1.33
Week 0 to 4	1.09<br>0.95,1.25	1.08<br>0.94,1.25	1.08<br>0.93,1.25	1.01<br>0.77,1.33	0.97<br>0.73,1.28	0.98<br>0.73,1.32	0.93<br>0.59,1.48	0.82<br>0.51,1.34	0.91<br>0.56,1.49	1.11<br>0.92,1.33	1.10<br>0.91,1.33	1.10<br>0.91,1.34
Week 5 to 8	0.98<br>0.86,1.12	0.98<br>0.85,1.13	1.00<br>0.86,1.15	1.00<br>0.77,1.30	0.99<br>0.76,1.29	1.00<br>0.76,1.32	0.85<br>0.54,1.33	0.82<br>0.51,1.31	0.91<br>0.56,1.46	0.93<br>0.77,1.11	0.93<br>0.77,1.13	0.95<br>0.78,1.16
Week 9 to 12††	0.97<br>0.78,1.20	0.97<br>0.78,1.21	1.00<br>0.79,1.27	0.97<br>0.63,1.50	0.93<br>0.60,1.46	1.02<br>0.64,1.64	0.68<br>0.29,1.58	0.62<br>0.25,1.56	0.72<br>0.28,1.82	1.00<br>0.75,1.32	0.99<br>0.74,1.33	1.02<br>0.75,1.40

*†‡§ Congenital heart defects: see definitions in Table 4.

‖ Individuals with missing values of covariates were excluded in the adjusted analyses, see numbers in Table 4

Relative risk (RR) with 95% confidence interval (CI) comparing supplement use with no use (reference) 4 weeks before to 8 weeks after conception (upper panel) or comparing initiation of folic acid supplements with no use/non-folic acid supplements (reference) 4 weeks before to 12 weeks after conception (lower panel). Model 1: RRs adjusted for country, year of birth, maternal age (years <=24, 25-29, 30-34, >=35), birth order (1, 2, 3+), maternal epilepsy before conception (yes/no), maternal body mass index (BMI) (<20, 20-24, 25-29, 30-34, =<35), maternal heart defect (yes/no). Model 2: RRs adjusted for covariates as in Model 1, in addition, maternal socioeconomic status (Denmark: level 1, 2, 3, 5, 6, 7) and maternal education (Norway: years <12, 12, 13-16, >=17). Model 3: RRs adjusted for covariates as in Model 1, in addition, maternal pregestational diabetes (yes/no), maternal smoking before conception (daily, no), maternal alcohol consumption 3 months before conception (yes/no).

** The four exposure categories in supplement use collapsed into no/yes; “no” is no folic acid use (no supplements and other supplements, no folic acid), and “yes” is folic acid use (folic acid only and folic acid plus other supplementation).

††In DNBC, including week 11.

Table S5. Relative risk (RR) of congenital heart defects* (overall), by maternal intake of folic acid supplements in the periconceptional period, in sensitivity analyses with restrictions to live births, singleton births, mother without heart defect, or planned pregnancies, combining 94,228 births in The Danish National Birth Cohort (DNBC), Denmark, 1996-2003, and 102,985 births in The Norwegian Mother and Child Cohort Study (MoBa), Noway, 1999-2009.

	Live births N=186,257‖ 2,092 births with heart defect			Singletons N=179,514‖ 1,957 infants with heart defect			Mother without heart defect N=185,818‖ 2,104 infant with heart defect			Planned pregnancy N=157,382‖ 1,797 infants with heart defect		
	No.	Adjusted RR# 95% CI		No.	Adjusted RR# 95% CI		No.	Adjusted RR# 95% CI		No.	Adjusted RR# 95% CI	
Supplement use (4 weeks before to 8 weeks after conception)												
None	563	1	Reference	520	1	Reference	654	1	Reference	445	1	Reference
Other supplements, no folic acid	103	1.00	0.81,1.23	95	0.99	0.80,1.23	104	1.00	0.81,1.23	86	1.06	0.85,1.34
Folic acid only	274	1.09	0.94,1.27	255	1.09	0.94,1.28	276	1.08	0.93,1.25	243	1.11	0.94,1.30
Folic acid plus other supplementation	1,152	1.06	0.96,1.18	1,087	1.09	0.98,1.22	1,160	1.06	0.96,1.18	1,023	1.07	0.96,1.21
Initiation of folic acid** (4 weeks before to 12 weeks after conception)												
None	572	1	Reference	523	1	Reference	574	1	Reference	460	1	Reference
Week -4 to -1	738	1.11	0.99,1.24	682	1.13	1.01,1.27	740	1.10	0.98,1.23	706	1.10	0.98,1.24
Week 0 to 4	339	1.08	0.94,1.24	324	1.13	0.98,1.30	344	1.08	0.95,1.24	296	1.07	0.92,1.24
Week 5 to 8	349	0.97	0.85,1.11	336	1.01	0.88,1.16	352	0.97	0.85,1.11	264	0.95	0.82,1.11
Week 9 to 12††	94	0.97	0.73,1.11	92	1.02	0.82,1.27	94	0.95	0.77,1.18	71	0.92	0.72,1.18

*Any congenital heart defect: see definitions in Table 4.

‖ Individuals with missing values of covariates were excluded in the adjusted analyses (10,339 among 196,596 live births; 9,986 among 189,500 singletons; 10,310 among 196,128 births of mothers without heart defect; 2,939 among 160,321 planned births).

Relative risk (RR) with 95% confidence interval (CI) comparing supplement use with no use (reference) 4 weeks before to 8 weeks after conception (upper panel) or comparing initiation of folic acid supplements with no use/non-folic acid supplements (reference) 4 weeks before to 12 weeks after conception (lower panel). RRs adjusted for country, year of birth, maternal age, birth order, maternal epilepsy, maternal BMI, maternal heart defect.

** The four exposure categories in supplement use collapsed into no/yes; “no” is no folic acid use (no supplements and other supplements, no folic acid), and “yes” is folic acid use (folic acid only and folic acid plus other supplementation).

†† In DNBC, including week 11.

Supplemental References:

1. Shaw GM, O'Malley CD, Wasserman CR, Tolarova MM and Lammer EJ. Maternal periconeptional use of multivitamins and reduced risk for conotruncal heart defects and limb deficiencies among offspring. *American Journal of Medical Genetics*. 1995;59:536-545.

2. Shaw GM, Carmichael SL, Yang W and Lammer EJ. Periconceptional nutrient intakes and risks of conotruncal heart defects. *Birth Defects Res A Clin Mol Teratol*. 2010;88:144-51.

3. Werler MM, Hayes C, Louik C, Shapiro S and Mitchell AA. Multivitamin supplementaion andr risk of birth defects. *American Journal of Epidemiology*. 1999;150:675-682.

4. Scanlon KS, Ferencz C, Loffredo CA, Wilson PD, Correa-Villasenor A, Khoury MJ and Willett WC. Preconceptional folate intak and malformations of the cardiac outflow tract. Baltimore-Washington Infant Study Group. *Epidemiology*. 1998;9:95-98.

5. Malik S, Cleves MA, Honein MA, Romitti PA, Botto LD, Yang S, Hobbs CA and National Birth Defects Prevention S. Maternal smoking and congenital heart defects. *Pediatrics*. 2008;121:e810-6.

6. Correa A, Gilboa SM, Botto LD, Moore CA, Hobbs CA, Cleves MA, Riehle-Colarusso TJ, Waller DK, Reece EA and National Birth Defects Prevention S. Lack of periconceptional vitamins or supplements that contain folic acid and diabetes mellitus-associated birth defects. *Am J Obstet Gynecol*. 2012;206:218 e1-13.

7. Czeizel A and Dudas I. Prevention of the first occurence of neural tube defects by periconceptional vitamin supplementation. *New England Journal of Medicine*. 1992;327:1832-1835.

8. Czeizel AE. Prevention of congenital abnormalities by periconceptional multivitamin supplementation. *BMJ*. 1993;306:1645-8.

9. Csaky-Szunyogh M, Vereczkey A, Kosa Z, Gerencser B and Czeizel AE. Risk and protective factors in the origin of conotruncal defects of heart--a population-based case-control study. *Am J Med Genet A*. 2013;161A:2444-52.

10. Bower C, Miller M, Payne J and Serna P. Folate intake and the primary prevention of non-neural birth defects. *Aust N Z J Public Health*. 2006;30:258-61.

11. van Beynum IM, Kapusta L, Bakker MK, den Heijer M, Blom HJ and de Walle HE. Protective effect of periconceptional folic acid supplements on the risk of congenital heart defects: a registry-based case-control study in the northern Netherlands. *Eur Heart J*. 2010;31:464-71.

12. Obermann-Borst SA, Isaacs A, Younes Z, van Schaik RH, van der Heiden IP, van Duyn CM, Steegers EA and Steegers-Theunissen RP. General maternal medication use, folic acid, the MDR1

C3435T polymorphism, and the risk of a child with a congenital heart defect. *Am J Obstet Gynecol*. 2011;204:236 e1-8.

13. Li X, Li S, Mu D, Liu Z, Li Y, Lin Y, Chen X, You F, Li N, Deng K, Deng Y, Wang Y and Zhu J. The association between periconceptional folic acid supplementation and congenital heart defects: a case-control study in China. *Prev Med*. 2013;56:385-9.

14. Leirgul E, Gildestad T, Nilsen RM, Fomina T, Brodwall K, Greve G, Vollset SE, Holmstrom H, Tell GS and Øyen N. Periconceptional Folic Acid Supplementation and Infant Risk of Congenital Heart Defects in Norway 1999-2009. *Paediatr Perinat Epidemiol*. 2015;29:391-400.
